# Advances in Molecular Imprinting Technology for the Extraction and Detection of Quercetin in Plants

**DOI:** 10.3390/polym15092107

**Published:** 2023-04-28

**Authors:** Kexi Ye, Shufang Xu, Qingqing Zhou, Sitao Wang, Zhigang Xu, Zhimin Liu

**Affiliations:** Faculty of Science, Kunming University of Science and Technology, Kunming 650500, China

**Keywords:** molecular imprinting technology, molecularly imprinted polymers, plant samples, quercetin

## Abstract

Quercetin is a kind of flavonoid compound, which has antioxidative, anti-aging and anti-cancer effects, so it is of great importance to study the efficient extraction and highly sensitive detection of quercetin. Molecular imprinting technology has remarkable selectivity and resistance to complex matrix interference, which is often used for extracting quercetin. The methods of molecular imprinted solid phase extraction, molecularly imprinted microsphere extraction, molecularly imprinted electrochemical sensor recognition and molecularly imprinted composite material extraction of quercetin from plant samples were discussed in detail. This review provides valuable information on efficient and sensitive methods for separating and purifying quercetin in plants. It also provides a technical reference for further investigation of the separation and analysis of active ingredients in natural products.

## 1. Introduction

Quercetin, a natural polyhydroxy flavonoid, is discovered in the leaves, flowers buds, seeds and fruits of many plants. Quercetin is one of the active components in many commonly used Chinese herbal medicines and natural products, such as honeysuckle, notoginseng, and ginkgo [1]. Quercetin has anti-bacterial, anti-oxidative, anti-tumor, and anti-inflammatory properties. Besides, it plays a pivotal role in the prevention and treatment of diabetes and the protection of neurons and the liver [2,3]. However, quercetin has a low content in natural products, a similar structure to other flavonoids and a complex sample matrix, which making its separation from plant samples extremely difficult. Therefore, exploring an efficient method for separating and determining quercetin in plant samples is crucial.

**Figure 1:**
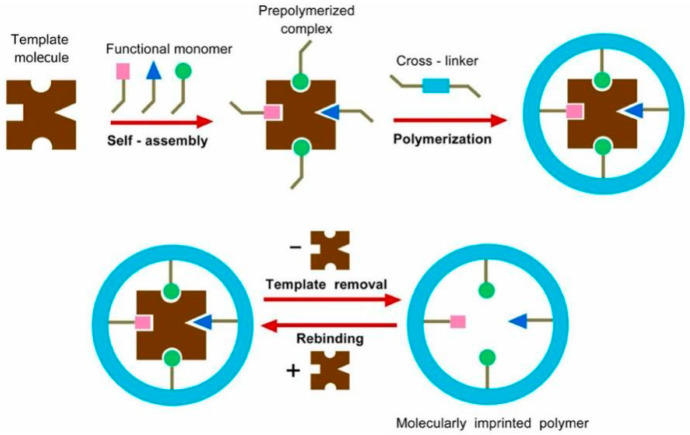
Schematic diagram of synthesis of molecularly imprinted polymers. Reproduced with permission from Ref. [5]. Copyright 2022, Elsevier.

Molecular imprinting technology (MIT) has been widely used to separate trace components in complex matrices. MIT is one of the powerful techniques for designing artificial receptors with specific specificity and selectivity toward targets, which can be employed as desirable materials in varieties of fields. Molecularly imprinted polymers (MIPs) are polymer substrates that can specifically recognize template molecules by simulating antigen-antibody or substrate-enzyme interactions. MIPs are synthesized via MIT. The combination of template molecules with functional monomers to form complexes is the commonly used method for preparing MIPs. Then, crosslinkers and initiators are added to the obtained product. After the removal of the target molecule from the resulting product, the MIPs and the template molecule will complement each other in space, shape, and functional groups, thus enhancing the selectivity of MIPs to identify imprinted molecules in complex samples [4]. The schematic diagram of the MIP preparation is displayed in Figure 1 [5]. MIPs have some advantages, such as resistance to harsh environments, low cost, good physicochemical stability, and reusability [6,7]. As a result, MIPs have attracted considerable attention in clinical drug analysis, solid-liquid extraction, chromatographic separation, bionic sensors, and other fields [8]. Moreover, MIT has been widely used in the extraction and analysis of active components in natural products with improved separation efficiency and accuracy [9,10].

This review focused attention on the application of multifarious MITs for extracting and analyzing quercetin in plant samples. Specifically, we focused on the extraction of quercetin by molecularly imprinted solid phase extraction, molecularly imprinted microsphere extraction, molecularly imprinted electrochemical sensors, and several special MIP materials. Compared with traditional separation methods of active components, such as ultrasonic extraction, reflux extraction, steam distillation, and high-speed countercurrent chromatography, MIT has good selectivity and improved separation efficiency of quercetin in plants. While Zhang et al. [9] focused on the application of MIPs to multifarious active substances in plants, such as organic acids, flavonoids and alkaloids, phenols, etc. Yang et al. [10] focused on the preparation of MIPs of flavonoids, covering precipitation polymerization, bulk polymerization, emulsion polymerization, surface imprinting and their applications. Finally, this review provides insight into the separation and purification of other chemical components in natural products.

## 2. Extraction and Analysis of Quercetin from Plant Samples Using MIPs

### 2.1. Extraction of Quercetin from Plant Samples by Molecularly Imprinted Solid-Phase Extraction

Traditional procedures for isolating and extracting quercetins from plant extracts are often solvent dependent and time consuming. There are many different methods for the extraction and determination of quercetins, covering spectrophotometry, high pressure liquid chromatography, capillary electrophoresis, luminescence, spectrophotometry and electrochemical sensors. These methods offer great experimental results, but fussy sample preparation is requested to eliminate the interference in the sample matrix. Thus, sample preparation ought to be operated to separate the analyte from the sample matrix. Solid phase extraction (SPE) is a technique that applies adsorbents to extract target molecules from a given sample. However, most materials applied for SPEs are not selective to analytes. To solve this problem, practical selective materials such as MIPs are developed.

Molecularly imprinted solid-phase extraction uses MIPs as sorbents and overcomes the shortcomings of poor specificity of traditional solid-phase extraction. It has the advantages of simple preparation, good stability, and reusability [11]. Molecularly imprinted solid-phase extraction can selectively recognize specific compounds or their structural analogs from complex matrices while preconcentrating the target analyte. It is attractive to concentrate and purify quercetin using MIPs solid phase extraction [12,13,14].

MIPs were prepared using acrylamide as functional monomer, quercetin as template molecule, tetrahydrofuran (THF) as pore source, and ethylene glycol dimethacrylate as crosslinking agent by thermal polymerization [15]. Two compounds with similar structures to quercetin, catechol and rutin, were used to test the selective recognition by the proposed MIPs. The results showed that MIPs had stronger selective binding ability for quercetin. On this basis, MIPs were used as adsorbent for solid phase extraction (SPE) to extract and enrich quercetin from guava samples, and HPLC-UV analysis was performed. MIPs with stereoselectivity and high affinity for quercetin in solid phase extraction provide a new method for the enrichment and determination of flavonoids in natural products.

Rahimi et al. [16] prepared a new quercetin MIP solid phase microextraction (SPME) fiber with good thermodynamic properties was on the surface of stainless steel wire by sol-gel method. Stainless steel was used as a low-cost and high-strength surface compared to conventional SPME fibers (fused silicon rods) to overcome the vulnerability of these fibers. Using tetraethyl orthosilicate as crosslinking agent and 3-amino-propyl triethoxy silane as functional monomer, the molecularly imprinted polymer was prepared by treating SS wire surface. The fiber was successfully used for the selective extraction and determination of quercetin in beverage samples with good extraction efficiency and high selectivity. The current sol-gel coating is very simple and easy to use in the preparation of stainless steel wire as MIP-SPME fiber. Compared with other commonly used MIP synthesis methods, this method has the advantages of cheap, fast and stable. The method has recovery rates between 94.92% and 98.00% for 0.1 μg/mL black tea, green tea, and coffee. In addition to the target compound as a template molecule, the target molecule-metal complex is always employed as a template for traditional molecular polymers. For example, Fan et al. [17] prepared metal coordination MIPs in strong polar solution methanol using the complex of quercetin and Zn (II) as a template and 4-vinylpyridine as a functional monomer. The imprinted polymer exhibited improved specific recognition and selectivity for quercetin-Zn (II) complexes. The adsorption selectivity of quercetin structure analogues rutin and naringin was poor, with separation factors ($\alpha_{1}$) of 3.21 and 1.91. Static allocation coefficient *K* and separation factor $\alpha_{1}$ are defined as:

(1) $$K=C_{P}/C_{s}$$

(2) $${\alpha_{1}=K}_{i}/K_{j}$$

where $C_{s}$ is the equilibrium concentration of substrate in solution (mmol/L), $C_{P}$ is the concentration of polymer-bound substrate (μmol/g), *j* and *i* are substrate molecules and template molecules respectively. When *i* = *j*, α= 1.

This method has a promising application prospect in the detection and separation of quercetin, the active ingredient of traditional Chinese medicine. Meanwhile, studying the role of metal coordination in molecular recognition will also contribute to the understanding of the processes and mechanisms of molecular imprinting and biometrics.

In addition, Pakade et al. [18] researched the influence of temperature on the extraction of quercetin from MIP. In order to form molecularly imprinted polymers with high recognition to quercetin molecules at high temperature, MIPs targeting quercetin molecules were prepared in different solvent systems using 4-vinylpyridine and ethylene dimethacrylate as raw materials. MIP prepared from THF/H_2_O/MeOH solvent mixture has a broad application prospect in the determination of quercetin in plant (onion). The binding ability of MIPs was compared at 25 °C and 84 °C. The slope of extraction time showed that mass transfer of analyte was higher at 84 °C than at 25 °C. In addition, at 84 °C, the MIP with the best extraction effect and its relevant NIP have higher binding capacity. The binding ability of MIP at 25 °C and 84 °C is 30 μmol/g and 120 μmol/g, respectively, while that of NIP at 25 °C and 84 °C is 15 μmol/g and 90 μmol/g, respectively. The selectivity of MIP was demonstrated at higher temperatures using a standard solution of the selected flavonols, showing that MIP still reserved its selectivity for quercetin. Analogous selectivity was obtained in the elementary application study of yellow onion aqueous extract.

For the purpose of expanding the application of MIPs, surface molecular imprinting as a new method has attracted great attention. In short, the so-called surface molecular imprinting is to take certain measures to limit nearly all binding sites to the surface with great approachability, so as to facilitate the recombination and removal of template molecules. Therefore, this way is especially appropriate for the imprinting of biological macromolecules. Petrova et al. [19] proposed molecularly imprinted membranes on the surface of silica microparticles, which is an organic monolithic polymer prepared by glutathione and 3-methylacroxypropyltrimethoxy-silane (gamma-Maps) in a thiol-ene cleavage reaction triggered by the heating of azodiisobutanitrile. Quercetin exists as a template. The surface of SiO_2_ particles was first modified with hydrolyzed γ-MAPS, and then the mercaptoene clicking reaction was performed in the presence of quercetin. The second method is to obtain an organic mineral polymer by clicking mercaptoene in solution, then hydrolyze γ-MAPS with acid or base, and then fix the product to the surface of silica particles. The results show that, compared with the method of obtaining MIP on the modified silica surface, the method of obtaining MIP in solution and fixing MIP on the silica surface provides a more effective molecular imprinting method.

Hong et al. [20] prepared quercetin MIP by surface molecular imprinting with SiO_2_ as the core. Then, quercetin was selectively extracted from Herba Lysimachiae samples using MIP as substrate solid phase dispersion (MSPD) adsorbent. Compared with other methods, the MSPD method completes extraction and cleaning in one step, reducing analysis time and solvent consumption. The MIP-MSPD method can be used for the determination of quercetin in complex drug samples by combining the high selectivity of MIP and the good clarification of MSPD for complex solid samples.

### 2.2. Extraction of Quercetin from Plant Samples by Molecularly Imprinted Microsphere Extraction

The traditional MIPs is simple to prepare, but the morphology of the material is irregular and the selective adsorption efficiency is low after embedding the effective imprinted spots. However, surface MIPs such as molecularly imprinted microspheres can effectively solve these problems [21,22]. The microspheres are more regular and have a larger specific surface area, higher adsorption and separation efficiency than other molecularly imprinted materials, and high selectivity. MIP microspheres are a new type of adsorption material obtained through MIP modification on the surface of some specific micron-nanoparticles, such as Fe_3_O_4_ magnetic nanoparticles and carbon nanotubes. The micron-nanoparticles are more regular and have a larger specific surface area than traditional materials, improving the adsorption and separation efficiency of MIPs. However, there are still some problems concerning the preparation method and molecular recognition effect of MIP microspheres that need to be solved: (1) There are few types of functional monomers and crosslinkers for preparing microspheres. Functional monomers mainly include methacrylic acid, 2- (trifluoromethyl) acrylic acid, crylic acid, 4-vinylpyridine. There are only a few crosslinking agents such as ethylene glycol dimethacrylate. Therefore, it is urgent to synthesize more special functional monomers and crosslinking agents to greatly expand the application range of molecularly imprinted microspheres. (2) At present, most molecularly imprinted microspheres are synthesized with liposoluble template molecules in the organic phase. For water-soluble target molecules, although the molecularly imprinted microspheres can be synthesized by reverse-phase suspension polymerization, their water absorption and swelling properties are relatively high and their rigidity is insufficient, so their molecular recognition performance is affected to some extent. How to obtain the molecularly imprinted microspheres of water-soluble molecules with certain rigidity and molecular recognition ability needs further research. (3) Although there are many researches on the synthesis of MIP microspheres, further studies are needed to realize industrialization and commercialization. For example, the application of chiral separation technology of drugs into large-scale production, the extraction of a large number of active ingredients in traditional Chinese medicine, and the reduction of preparation cost are all topics worthy of further study.

Magnetic nanomaterials have been successfully introduced into MIPs. Magnetic MIPs (MMIPs) can specifically detect the target component and quickly separate it from the matrix under an external magnetic field. In previous studies, quercetin can be adsorbed on different nanomaterials, but it lacks selectivity for specific analytes. In order to improve the selectivity and adsorption capacity of quercetin, high performance MIPs modified with magnetic graphene oxide (MGO) were prepared by coprecipitation method [23]. MGO/MIP has a high load (369 mg/g) and selective capacity, making the performance of the nanomaterial better than previously reported. Quercetin was determined by magnetic solid phase extraction and high performance liquid chromatography, and the extraction conditions were studied. Under optimized conditions, MGO-MIP showed good performance and high sensitivity in the detection of quercetin and its similar molecules in green tea and serum samples.

A core-shell magnetic MIPs for adsorption and recognition of quercetin was prepared by combining surface imprinting technology with nanotechnology [24]. The obtained Fe_3_O_4_@MIPs has high adsorption capacity and selectivity for quercetin. By using an external magnetic field, they can be easily separated from a complex matrix. In addition, Fe_3_O_4_@MIPs can be successfully used for the specific separation and determination of quercetin in apple samples with high recovery (89.2–93.6%). Therefore, this method (Fe_3_O_4_@MIPs) can be considered as a promising and practical method for the highly selective separation and determination of quercetin in fruits.

Furthermore, Ruan et al. [25] prepared MIPs using surface MIT. The prepared MIPs had a specific recognition effect on quercetin. The characterization results showed that the MIPs were successfully deposited on the surface of the Fe_3_O_4_ magnetic carrier. The adsorption selectivity of quercetin using MIPs was higher than that of quercetin using magnetic non-imprinted polymers. Moreover, the polymers had two different binding sites, with maximum adsorption capacities of 23.041 and 29.923 mg/g.

MIPs and graphene quantum dots (GQDs) are smart materials in green analytical chemistry and have a wide range of applications. Greening in analytical chemistry is mainly about reducing the use of solvents and harmful chemicals. Green analytical chemistry is part of the concept of sustainable development. Miniaturization of analyzers, shortening analysis time and obtaining reliable analysis results are important aspects of green analytical chemistry.

**Figure 2:**
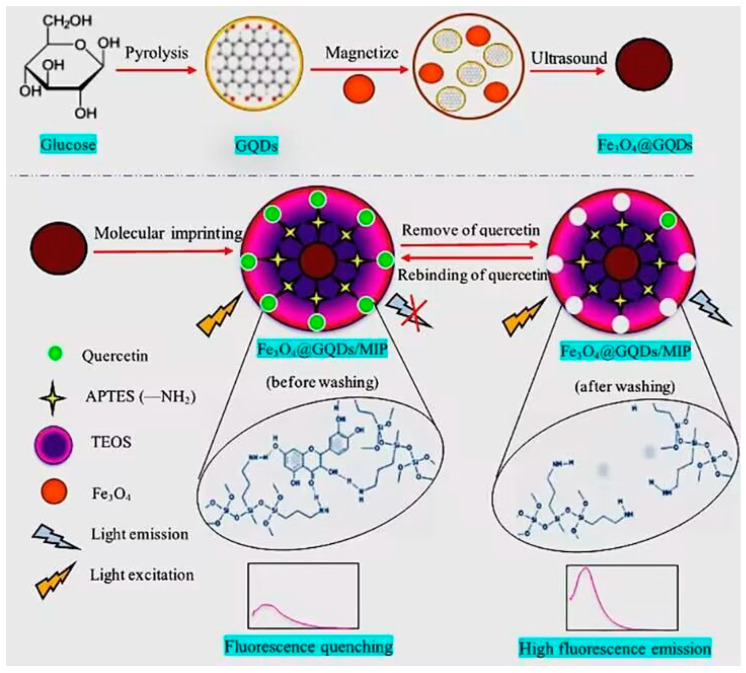
Synthesis process diagram of Fe_3_O_4_@GQDs/MIP (before and after washing). Reproduced with permission from Ref. [26]. Copyright 2023, Elsevier.

Mantashloo et al. [26] used the self-assembly reaction of quercetin molecules with triethoxysilane to synthesize a new type of green adsorbent, magnetic GQDs modified with MIP coatings (Fe_3_O_4_@GQDs/MIP) (Figure 2). The appearance of quercetin results in the interaction of molecules with Fe_3_O_4_@GQDs/MIP and interference with the electronic properties of GQDs, which leads to the quenching of fluorescence emission. In accordance with Stern-Volmer equation (F_o_/F = 1 + Ksv·[C]), fluorescence intensity has a linear relationship with concentration. Thus, the reduction of fluorescence emission signal (ΔF) can be used to quantitatively measure quercetin. The adsorbent had high selectivity, high sensitivity, and low cost, which could effectively analyze quercetin medicinal compounds. The green adsorbent effectively detected green tea, cumin, and thyme, with a recovery of 97.61–102.11% and relative standard deviation of 1.18–3.71%. MMIPs combined magnetic nanoparticles and MIPs to enhance adsorption selectivity and realize rapid separation.

MIP was synthesized rapidly based on the green technology of high energy ultrasonic irradiation [27]. Combined with fluorescence method, the selective SPE and sensitivity measurement of quercetin were realized effectively. It has good sensitivity and selectivity to quercetin. In addition, the proposed MMIP was integrated successfully with the developed paper-based analytical devices for field smartphone analysis of quercetin. In addition, the developed MIP has the advantages of simple, rapid preparation, high selectivity, good stability and low cost, which confirms that MIP can be employed as an excellent synthetic antibody for the development of new biosensors for the detection of a variety of analytes in plant samples.

The selective MIP of quercetin was successfully synthesized by a straightforward ultrasonic-assisted coprecipitation polymerization method [28]. Ultrasonic energy is not only green energy, but also the polymerization time is much shorter than the traditional route. Magnetic nanoparticles were combined with the polymerization network, the target analytes were inserted into the polymerization reaction, and specific binding sites of similar shape and size were left after the target analytes were extracted. The developed magnetite molecularly imprinted polymer has the advantages of high selectivity, large adsorption ability, good repeatability, simple preparation, simple operation, green energy utilization, low solvent consumption, low cost, and good reproducibility. A good linear concentration range (0.32–25µg/mL) was observed. LOD and LOQ were 0.06 µg/mL and 0.2 µg/mL, respectively. The adsorbent was successfully used for selective extraction of quercetin from onion samples [28].

### 2.3. Recognition of Quercetin from Plant Samples by Molecularly Imprinted Electrochemical Sensor 

Molecularly imprinted sensing technology has attracted considerable attention owing to its high sensitivity, rapid response, simple analysis, real-time monitoring, and low cost [29]. Common sensing techniques used in this field include electrochemical sensing [30,31], optical sensing [32,33], and surface plasmon resonance sensing [34,35,36]. Quercetin is mainly identified using a molecular imprinting electrochemical sensor.

Liu et al. [37] prepared molecularly imprinted films with quercetin as a template molecule and o-aminophenol as a functional monomer through electropolymerization on the surface of a gold electrode. Single-walled carbon nanotubes (SWNTs)/graphene (GR)/Au electrode was subjected to CV scanning at a speed of 0.1 V/s in 1.0 × 10^−3^ mol/L o-aminopol and 6.67 × 10^−5^ mol/L quercetin at a potential range of −0.1–+0.8 V, and MIP/SWNTs/GR/Au electrode was prepared by electropolymerization. The MIP/SWNTs/GR/Au electrodes were eluted for 10 min with a mixture of aqueous ethanol and distilled water (aqueous ethanol: distilled water = 2:1, *v*/*v*), and a molecular imprinted sensor with specific hole recognition was obtained. It was used to specifically recognize quercetin. Rutin, similar to quercetin in structure, was used for the control experiment. The result showed that the imprinted films had better selectivity for quercetin. In addition, the sensors exhibited good stability and repeatability. The recovery rate was approximately 97.8–104.0%. Liu et al. [38] performed a similar experiment by studying the response of a gold electrode to quercetin. They prepared molecularly imprinted films through electrochemical polymerization of o-aminophenol, which could selectively recognize quercetin. Electropolymerization was carried out in 0.001 mol/L o-aminophenol and 0.0001 mol/L quercetin solutions with pH 5.5. During the 30-week scanning process, the current intensity gradually decreased with the increase of scanning times, indicating that the electrochemical polymerization process of o-aminophenol and quercetin on the electrode was irreversible. A very dense non-conductive polymer film was formed on the surface of Au electrode, which generated an obvious reduction in the oxidation amount of o-aminophenol on the electrode surface and a significant decrease in current. Moreover, rutin was used for a selective response experiment. The finding revealed that the sensors had better selectivity for quercetin. The response was fast, and LOD was 2.0 × 10^−6^ mol/L with a linear range of 6.0 × 10^−6^–1.0 × 10^−4^ mol/L, providing a simple and accurate method for detecting quercetin.

**Figure 3:**
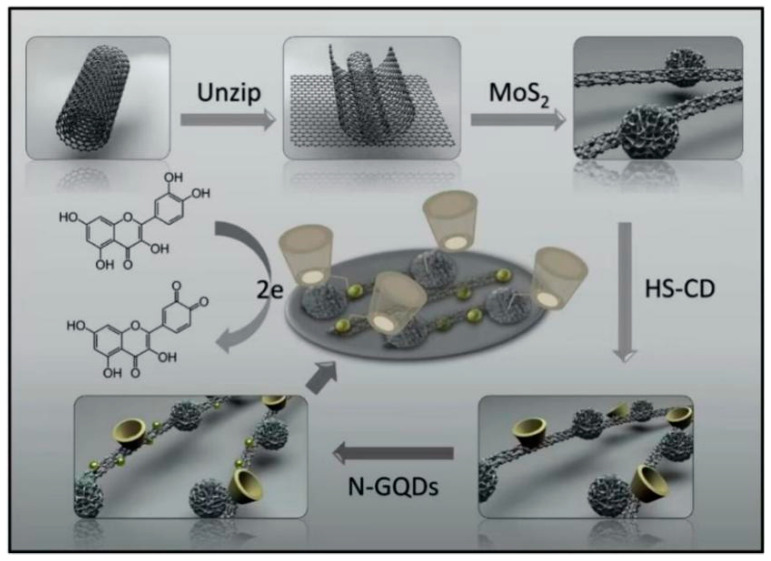
Schematic illustration of the outstanding electrochemical redox process of quercetin on the molybdenum disulfide-carbon nanotube @ graphene oxide nanoribbons/per-6-deoxy-(6-thio)-cyclodextrin/graphene quantum dots/glassy carbon electrode. Reproduced with permission from Ref. [39]. Copyright 2019, Elsevier.

Moreover, a MoS_2_ multi-walled carbon nanotube @ graphene oxide nanoribbons (MoS_2_-CNTs@GONRs) with excellent electrocatalytic and electrochemical properties were successfully synthesized [39]. Moreover, an ultra-sensitive quercetin electrochemical sensor was successfully developed based on MoS_2_-CNTs@GONRs, per-6-deoxy-(6-thio)-β-cyclodextrin (HS-CD) and amino-functionalized graphene quantum dots (N-GQDs) (Figure 3). On account of the strong catalytic characters of MoS_2_, the remarkable electrochemical property of CNTs@GONRs, the great stability and biocompatibility of N-GQDs and the good enrichment ability of HS-CD, this sensor has indicated prominent detection performance for quercetin. Furthermore, the sensor displayed great stability and accuracy in the determination of real samples.

Furthermore, Yao et al. [40] invented an electrochemical sensor for detecting quercetin by incorporating magnetic reduced graphene oxide into MIPs on the surface of a screen-printed electrode. Under the optimum conditions, the linear response of the modified electrode ranged from 20 to 250 μmol/L. Modified electrodes (MIP/SPE/MrCO) exhibited higher sensitivity and selectivity due to the synergistic effect of particle binding. The modified electrode surface can be quickly refreshed by an applied magnetic field. Moreover, the electrode exhibited high selectivity, reliability, stability, reproducibility, and good determination effect of quercetin in spiked drug samples.

Qiu et al. [41] established a novel flow injection chemiluminescence (FI-CL) sensor using molecularly imprinted polymer microspheres (MIPm) as recognition element and Lumino-NaOH-H_2_O_2_ system for the determination of quercetin. Attributing to the specific binding site on the MIP microspheres, quercetin can be selectively absorbed, which enhances the sensitivity and selectivity of CL analysis. From this perspective, the CL sensor was prepared and used for the direct detection of quercetin in drug samples and no sample purification is required. Because MIPm can successfully weaken interference. The CL sensor offers a sensitive and rapid method for on-line detection of quercetin with content results.

Sun et al. [42] prepared MIPs by adding graphene oxide to polypyrrole film as substrate. Graphene oxide has a large surface to volume ratio and high electron transmittance, which can observably enhance the sensitivity of graphene-based electrochemical modified electrodes, and MIT can significantly improve the selectivity of modified electrodes. The electrode has good reproducibility and stability for the electrochemical detection of quercetin. In the linear range, rutin and morin with the same structure and concentration as quercetin, but did not affect with the determination of quercetin. This method is suitable for the analysis of quercetin in complex substrates.

Lu et al. [43] constructed a new molecularly imprinted electrochemical sensor for the sensitive determination of quercetin based on a glassy carbon electrode (GCE) modified with gold nanoparticles (AuNPs), β-cyclodextrin (β-CD) and graphene (GR). The MIPs were employed as the molecular recognition element, which were developed by electropolymerization employing pyrrole as a monomer and quercetin as a template. Quercetin was selected as the target molecule. The preparation process is simple, and the prepared β-CD/AuNPs/GR composite requires no further chemical treatment, and is easy to purify, store and redisperse in water. Flavonoids usually found in Chinese herbs and have anti-allergy, anti-cancer, antiviral activities and anti-inflammatory. The shape and size of quercetin are accordant with the β-CD cavity. With the assistance of pyrrole, MIPs (MIPs/β-CD/AuNPs/GR/GCE) for establishing imprinted electrochemical sensors were produced by electropolymerization. MIPs/β-CD/AuNPs/GR/GCE showed high selectivity, rapid rebinding kinetics and good sensitivity under the synergistic effect of MIPs, β-CD, AuNPs and GR. Wang et al. [44] applied o-phenylenediamine as functional monomer and quercetin as template molecules. A novel method for the detection of quercetin by different differential pulse voltammetry was developed. This method has the merits of good selectivity, high sensitivity. This way is appropriate for the detection of quercetin in actual samples.

Quercetin molecular-imprinted film modified electrode was prepared by cyclic voltammetry using o-phenylenediamine as functional monomer and quercetin as template molecule. The spectral pure graphite rod was prepared into a wax impregnated graphite electrode (GE) and cleaned until clean. Using GE as the working electrode, a mixture of 1.00 × 10^−2^ mol/L OPD, 4.00 × 10^−4^ mol/L quercetin and 0.10 mol/L KCl as the modifier, the modified solution was obtained in the range of +1.50–−1.00 V potential. A continuous cycle voltammetry scan was performed for 30 cycles at a sweep speed of 0.05 V/s. The electrodes were removed, rinsed with water, and then placed in phosphate buffer solution with pH 7.0. After 30 cycles of scanning, polyo-phenylenediamine graphite modified electrode with quercetin imprinted was obtained and used for the detection of quercetin. In the buffer solution of HAc-NH_4_Ac (pH 4.0), the differential pulse voltammetry was employed as the electrochemical excitation signal. Quercetin produced a sensitive oxidation peak at 0.37 V, and the peak current showed a linear relationship with its concentration in the range of 8.00 × 10^−8^–1.00 × 10^−3^ mol/L. The detection limit was 5.00 × 10^−8^ mol/L. The new method was employed to the determination of quercetin in two kinds of ginkgo biloba drugs with recoveries ranging from 99.2% to 102%.

The characteristics of the above-mentioned sensors and four other molecularly imprinted electrochemical sensors are comprehensively compared in Table 1.

### 2.4. Extraction of Quercetin from Plant Samples by MIP-Based Composites of Materials Extraction

MIP composites have attracted considerable academic attention in their use for detecting quercetin and in other research areas. The MIP-based composite material can improve the specific recognition ability and selectivity of a sensor to a greater extent and inhibit the oxidation of quercetin, which is difficult to control using the above three methods. Currently, various composite materials, such as scaffolds, silica, and advanced dielectric materials, are used as adsorbent materials. In the pre-treatment of biological samples, the protein, humic acid and other biological macromolecules in the sample often cause interference to small molecular target compounds. In order to solve this problem, restricted-access material (RAM) is employed as SPE adsorbent. The difference between RAM-SPE materials and classical SPE materials is that RAM-SPE materials not only have functional groups for adsorption of target compounds, but also carry out hydrophilic modification on the outer surface of SPE materials, which can block interference macromolecules and prevent the irreversible degeneration of macromolecular proteins. The problem of column efficiency reduction caused by denatured proteins blocking SPE packing micropores is avoided. RAM materials have the following characteristics: (a) RAM materials have a specific exclusion barrier. Physical exclusion barrier, through the micropore diameter for the removal of macromolecules. A chemical barrier that resists macromolecules through a network of chemically synthesized polymers. (b) RAM materials have the biologically compatible outer layer. There is no irreversible interaction with the biomacromolecules in the sample. (c) RAM materials have the inner surface layer with adsorption function. The inner surface of the micropore of filler particles has reversed phase or ion-exchange functional groups, which can interact with small molecular compounds entering the micropore. Restricted-access MIPs are composite materials combining the advantages of restricted materials and MIT. Meanwhile, they can effectively reject proteins in various complex samples and are highly selective in separating and enriching target molecules. The restricted-access MIPs can be classified into the following types: (I) hydrophilic comonomers, (II) hydrophilic comonomers and BSA, (III) comonomers that became hydrophilic after treatment, and (IV) comonomers that became hydrophilic after treatment and BSA [45,46].

Li et al. [47] prepared MIPs using hexagonal boron nitride as a two-dimensional scaffold, quercetin as a template, ethylene glycol dimethyl acrylate as a crosslinking agent, methanol as a porogen, and azodiisobutyronitrile as an initiator. The quercetin detection ability of the new adsorbent was significantly enhanced compared with the traditional MIP particles, with a recovery rate of 97.5%.

Meanwhile, to enhance the detection property of MIPs, Liang et al. [48] grafted quercetin-MIPs onto the pore wall of an ordered macroporous thiol-functionalized silica to prepare ordered macroporous MIPs. Isothermal adsorption and kinetic experiments revealed that the polymers showed a higher selectivity and affinity for quercetin than analogues of quercetin, with an imprinting factor(I) of 1.79. Here, *K_p_* is define as

(3) $$K_{p}=\frac{S_{b}}{X_{f}}$$

where *X_f_* is the concentration of substrate remaining in solution after adsorption onto the polymer, *S_b_* is the amount of substrate bound to the polymer. *I* is defined as

(4) $$I=\frac{K_{pMIPs}}{K_{pNIPs}}$$

(5) $$IF=\frac{Q_{MIP}}{Q_{NIP}}$$

(6) $$\alpha_{2}=\frac{{IF}_{T}}{{IF}_{C}}$$

where *Q_NIP_* (mg/g) and *Q_MIP_* (mg/g) were the capacity for adsorption of *NIP* and *MIP* for quercetin, respectively. *IF_C_* and *IF_T_* were the imprinting factor for the competitive compound and the template molecule quercetin.

Moreover, the adsorption of quercetin in the particles was improved. Consequently, the available recognition sites of prepared polymers were greatly upgraded compared with those of the traditional MIPs. Additionally, when the macroporous MIPs were used to adsorb quercetin in the extract of Ginkgo biloba leaves through solid-phase extraction, the recovery rate was 55.1%, parallel with the elution recoveries of 17.4% and 3.2% obtained by extracting kaempferol and isorhamnetin. The result demonstrates the excellent selectivity of the polymers to quercetin. Moreover, Zhi et al. [49] prepared molecularly imprinted composite polymers with quercetin as a template and silica as a carrier. The maximum adsorption capacity of the composite polymers for quercetin was 35.70 mg/g. Compared with non-imprinted polymers, MIPs exhibited a high selectivity and specific detection for quercetin. The coefficient of selectivity ($\alpha_{2}$, Equations (5) and (6)) for quercetin was 1.61. MIPs retained more than 90% of their initial adsorption capacity after five regenerations of MIPs, indicating that the MIPs exhibit good selective adsorption for quercetin and excellent usability and have potential application prospects.

**Figure 4:**
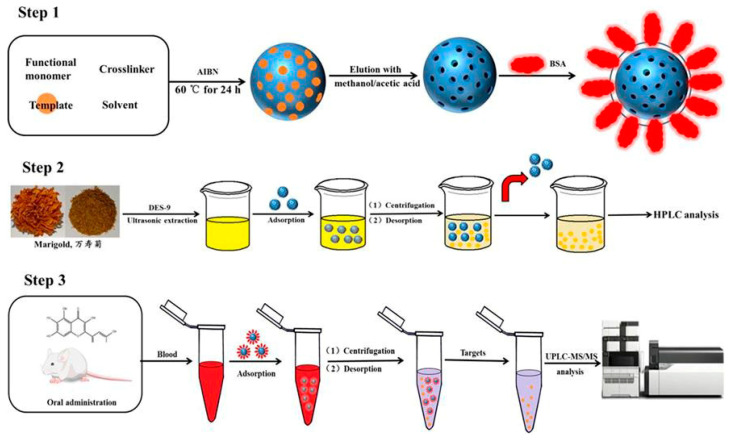
Schematic diagram of the synthesis and application of MIPs and RAMIPs. Reproduced with permission from Ref. [50]. Copyright 2022, Elsevier. Note: AIBN: Azobisisobutyronitrile; BSA: Bovine serum albumin; DES-9: Deep eutectic solvent-9; HPLC: High performance liquid chromatography; UPLC-MS/MS: Ultra-high performance liquid chromatography-mass spectrometer/mass spectrometer.

Additionally, quercetagetin based MIPs and restricted access molecularly imprinted polymers (RAMIPs) were synthesized to separately enrich quercetagetin from deep eutectic solvent (DES) extract of T. erecta flower and plasma for the first time [50]. The results of macromolecular rejection experiments showed that almost all the selected proteins could be completely rejected by the restricted pathway MIPs (RAMIPs), which revealed the outstanding macromolecular rejection property of RAMIPs. Its exclusion effect is higher than that of MIP. They were used to separately enrich quercetin from the deep eutectic solvent extract of T. sturgeon (Figure 4). Then, in the static, dynamic, and selective adsorption experiments, the adsorption capacity of traditional and restricted-access MIPs was 27.2 and 23.1 mg/g, respectively, and their imprinting factors (IF, Equation (5)) were 2.57 and 2.09, respectively. The main advantage of restricted-access MIPs is that they can almost reject all selected proteins compared with traditional polymers. Compared with the traditional precipitation method, the restricted-access MIPs can effectively characterize the absorption of quercetin.

The above three molecular imprinting methods for quercetin extraction were compared, as shown in Table 2.

## 3. Conclusions and Prospects

In recent years, MIT has been widely used to isolate quercetin from plant samples. At present, the emergence of novel molecularly imprinted adsorbent materials such as molecularly imprinted microspheres, molecularly imprinted electrochemical sensors, molecularly imprinted composite materials, etc., has improved the practicality, specificity detection ability and selectivity of molecularly imprinted adsorbent materials. Compared with traditional molecularly imprinted microspheres, magnetic molecularly imprinted microspheres have higher adsorption efficiency, molecularly imprinted electrochemical sensors have faster detection speed and excellent operability, and molecularly imprinted composite materials can integrate the advantages of several materials to improve the specific recognition ability and selectivity. Compared with other reviews, we focused on the extraction and detection of quercetin from plants by four molecular imprinting techniques. These new molecularly imprinted adsorbent materials make MITs more attractive for analyzing quercetin in plant samples.

However, the application of MIPs is limited due to short-term development. First, the crosslinkers, polymerization methods, and variety of functional monomers available are limited, thus limiting the application range. In the aqueous reaction system, the competition between template molecules and water impairs the non-covalent forces between functional monomers and template molecules, thus the application of MIPs in the separation of natural compounds is further limited. Second, poor template removal conditions may lead to poor impression results. The recognition mechanism, formation process and binding mechanism of template molecules are unclear; therefore, they need to be further studied.

With further advances in synthesis methods, biotechnology, and material science, the application of MIPs will be more extensive and satisfactory. First, computer simulation techniques focus on interpreting the intensity of template molecules’ interactions with functional monomers and solvents at the molecular level, which will contribute to theoretical and experimental studies of MIPs. Second, the research and development of new methods for preparing soluble MIPs will improve the adsorption capacity of MIPs, thereby facilitating industrial production.

## Figures and Tables

**Table 1:** Molecularly imprinted electrochemical sensors used for detecting quercetin.

Samples	Modified Electrode	Linearity Range /mol/L	LOD /mol/L	Recovery/%	Reference
Dark tea	MIP/SWNTs/GR/Au ^a^	3.00 × 10^−7^–1.92 × 10^−5^	1.00 × 10^−7^	97.8–104.0	[37]
Dark tea	MIP/Au ^b^	6.00 × 10^−6^–1.00 × 10^−4^	2.00 × 10^−6^	99.0–101.2	[38]
Juices	MIP/MoS_2_-CNTs@GONRs/HS-CD/GQDs/GCE ^c^	2.00 × 10^−9^–1.60 × 10^−6^	8.20 × 10^−10^	95.4–106.1	[39]
Pharmaceuticals	MIP/SPE/MrCO ^d^	2.00 × 10^−8^–2.50 × 10^−4^	1.30 × 10^−8^	99.5–104.3	[40]
Chemicals	MIP/Luminol-NaOH-H_2_O_2_^e^	1.40 × 10^−6^–1.60 × 10^−4^	9.30 × 10^−7^	98.1–100.0	[41]
Juices	MIP/GO/GC ^f^	6.00 × 10^−7^–1.50 × 10^−5^	4.80 × 10^−8^	97.4–101.4	[42]
Pharmaceuticals	MIP/β-CD/AuNPs/GR/GCE ^g^	1.00 × 10^−9^–1.00 × 10^−6^	1.00 × 10^−10^	97.6–102.1	[43]
Gingko drugs	MIP/OPD/GE ^h^	8.00 × 10^−8^–1.00 × 10^−3^	5.00 × 10^−8^	99.2–102.0	[44]

^a^: molecularly imprinted polymer/single-walled carbon nanotubes/graphene/Au; ^b^: molecularly imprinted polymer/Au; ^c^: molecularly imprinted polymer/MoS_2_/carbon nanotubes/graphene oxide nanoribbons/per-6-deoxy-(6-thio)-β-cyclodextrin/graphene quantum dots/glassy carbon electrode; ^d^: molecularly imprinted polymer/screen-printed electrode/magnetic reduced graphene oxide; ^e^: molecularly imprinted polymer/Luminol-NaOH-H_2_O_2_; ^f^: molecularly imprinted polymer/graphene oxide/glass carbon; ^g^: molecularly imprinted polymer/β-cyclodextrin/gold nanoparticles/graphene/glassy carbon electrode; ^h^: molecularly imprinted polymer/o-pheny-lenediamine/graphite electrode.

**Table 2:** Comparison of extraction methods of quercetin by molecular imprinting.

Methods	Type of Polymerization	Sample	Materials	Monomer; Crosslinker; Initiator; Template	Linearity Range /mol/L	LOD /mol/L	Recovery/%	Ref.
molecularly imprinted solid-phase extraction	thermal polymerization	cacumen, platycladi	MIP	acrylamide; EGDMA ^a^; AIBN; quercetin	−	−	80.21–89.15, 85.33–95.28	[15]
	sol-gel process	tea, coffee	MIP	APTES ^b^; AIBN; TEOS ^c^	0.05–100 μg/mL	9.94 ng/mL	94.20–98.50	[16]
	−	−	MIP	4-vinylpyridine; AIBN; complex of quercetin and Zn (II)	−	−	−	[17]
	−	yellow onion	MIP	4-vinylpyridine; EGDMA; AIBN; quercetin	−	−	−	[18]
molecularly imprinted microsphere extraction	co-precipitation technique	green tea, serum	MGO ^d^ -MIP	Methacrylic acid; EGDMA; AIBN; quercetin	0.001–3 μg/mL; 0.005–3 μg/mL	0.09 ng/mL; 0.70 ng/mL	82–100; 83–100	[23]
	surface imprinting technology	apple	magnetic Fe_3_O_4_@MIPs	APTES; EGDMA; AIBN;quercetin	1–400 μg/mL	0.20 μg/mL	89.2–93.6	[24]
	Surface molecular imprinting	quercetin	quercetin-MMIPs ^e^	N-vinylpyrrolidone and Acrylic Acid; N, N’-methylene diacrylamide; AIBN; quercetin	−	−	−	[25]
	−	green tea, cumin, thyme	FeO@GQDs ^f^/MIP	APTES; TEOS; AIBN; quercetin	5–220 ng/mL	0.54 ng/mL	97.61–102.11	[26]
	radical polymerization	orange juice, tea	MMIP-coated Fe_3_O_4_-Chitosan	MAA; EGDMA; AIBN; ammonium persulfate and quercetin	0.005–1.25 μg/mL	1.1 ng/mL	92.2–104.7	[27]
	ultrasonic mediated co-precipitation polymerization	onion	Fe_3_O_4_@SiO_2_@ NH_2_-quercetin-MIP	Methacrylic acid; EGDMA; AIBN; quercetin	0.32–25 μg/mL	0.06 µg/mL	96–98.6	[28]
MIP-based composites of materials extraction	−	−	h-BN-MIP nanoparticles	-; EGDMA; AIBN; quercetin	−	−	98.9–100.3	[47]
	surface imprinting method	gingko	OMMIPs ^g^	4-VP ^h^; EGDMA; AIBN; quercetin	−	−	−	[48]
	Sol-Gel Surface-MIP	−	−	APTES; TEOS; AIBN; quercetin	−	−	−	[49]
	precipitation polymerization	natural medicine, blood	−	2-VP ^i^; EDMA; quercetin	−	−	65.27–78.77	[50]

“−”: No mentioned. ^a^: ethylene glycol dimethacrylate; ^b^: APTES: 3-aminopropyltriethoxysilane; ^c^: tetraethyl orthosilicate; ^d^: magnetic graphene oxide; ^e^: Magnetic molecularly imprinted polymer; ^f^: graphene quantum dots; ^g^: Ordered macroporous imprinted polymers; ^h^: 4-Vinylpyridine; ^i^: 2-Vinylpyridine.

## Data Availability

There are no data associated with this publication.
